# 

**Published:** 1866-06

**Authors:** 


### CONTENTS.

### ORIGINAL CONTRIBUTIONS.

ART. XIX. Practical Remarks on Blood-Letting. By D. B. Trimble, M.D., Chicago, ---------- 321

ART. XX. Entropion and Trichiasis of the Upper Lid; their Radical Treatment, by an Operation, without Division of the Skin. By Jos. S. Hildreth, M.D., etc., ---------- 328

ART. XXI. The Primary Surgery of Gen. Sherman's Campaigns. By E. Andrews, M.D., Prof. of Surgery in Chicago Medical College, and J. M. Woodworth, M.D., Late Medical Director of the Army of the Tennessee, ---------- 332

### SELECTIONS.

Some Notes on Arrow-Wounds. By Elliott Coues, M.D., U.S.A., ---------- 350

On a Certain Form of Hæmoptysis, Unassociated with Pulmonary Tuberculosis. By Richard Payne Cotton, M.D., F.R.C.P. Lond., etc., ---------- 357

New and Ready Mode of Producing Anæsthesia, ---------- 360

Causes of Intermittent and Remittent Fever, ---------- 363

Process of Disinfection, ---------- 365

Statistics of the Profession in Paris, ---------- 366

### EDITORIAL.

American Medical Association, ---------- 367

New Books:—

Recent Advances in Ophthalmic Science. By H. W. Williams, Boston, 375

Asiatic Cholera. By F. A. Burrall, M.D., ---------- 375

Transactions of the American Medical Association, Vol. 16, for 1865, -- 375

The Aurora Homeopathic Quacks treated to an "Allopathic Dose of Facts," ---------- 376

Letter, and Prospectus of Leonardo da Vinci MS., ---------- 378

The Rinderpest, ---------- 380

Mortality of the City of Chicago for March, 1866, ---------- 381

